# A comparative analysis of human and mouse islet G-protein coupled receptor expression

**DOI:** 10.1038/srep46600

**Published:** 2017-04-19

**Authors:** Stefan Amisten, Patricio Atanes, Ross Hawkes, Inmaculada Ruz-Maldonado, Bo Liu, Fariborz Parandeh, Min Zhao, Guo Cai Huang, Albert Salehi, Shanta J. Persaud

**Affiliations:** 1 Diabetes Research Group, Division of Diabetes & Nutritional Sciences, Faculty of Life Sciences & Medicine, King’s College London, London, UK; 2 Department of Clinical Science, Division of Islet Cell Physiology, SUS, University of Lund, Malmö, Sweden

## Abstract

G-protein coupled receptors (GPCRs) are essential for islet function, but most studies use rodent islets due to limited human islet availability. We have systematically compared the GPCR mRNA expression in human and mouse islets to determine to what extent mouse islets can be used as surrogates for human islets to study islet GPCR function, and we have identified species-specific expression of several GPCRs. The A_3_ receptor (ADORA3) was expressed only in mouse islets and the A_3_ agonist MRS 5698 inhibited glucose-induced insulin secretion from mouse islets, with no effect on human islets. Similarly, mRNAs encoding the galanin receptors GAL_1_ (GALR1), GAL_2_ (GALR2) and GAL_3_ GALR3) were abundantly expressed in mouse islets but present only at low levels in human islets, so that it reads (GALR3) and galanin inhibited insulin secretion only from mouse islets. Conversely, the sst1 receptor (SSTR1) was abundant only in human islets and its selective activation by CH 275 inhibited insulin secretion from human islets, with no effect on mouse islets. Our comprehensive human and mouse islet GPCR atlas has demonstrated that species differences do exist in islet GPCR expression and function, which are likely to impact on the translatability of mouse studies to the human context.

It is well-established that secretion of hormones from islets of Langerhans is regulated by activation of islet cell G-protein coupled receptors (GPCRs) by neurotransmitters, paracrine actions of islet hormones themselves, excluding insulin which acts through a tyrosine kinase receptor, and by circulating hormones^1^. Thus, it has been known for many years that parasympathetic and sympathetic neurotransmitters act at specific muscarinic and adrenergic GPCR subtypes to potentiate and inhibit the stimulatory effects of nutrients on insulin secretion, to allow fine-tuning of the insulin secretory response^2^. In addition, glucagon stimulates insulin and somatostatin release, while somatostatin inhibits glucagon and insulin release^3^. Furthermore, GLP-1, an incretin released from the gastrointestinal tract following food intake, acts at GPCRs on islet β- and α-cells to stimulate insulin and inhibit glucagon secretion^4^, and GIP, another incretin, also potentiates glucose-induced insulin release. The GLP-1 receptor is probably the most well characterised of all islet GPCRs, and several GLP-1 receptor agonists and DPP4 inhibitor drugs that stabilise incretin levels are in widespread clinical use as therapies for type 2 diabetes (T2D)^5^. A number of other GPCRs, including GPR119, FFAR1, GPRC5B and GPRC5C, all of which are expressed by human islets^1^, have also emerged as drug target candidates for the treatment of T2D^6^,^7^.

In addition to the reasonably well-characterised GPCRs, some of which are outlined above, human islets express almost 300 additional GPCRs^1^,^8^,^9^, but most of these have poorly characterised roles in islet physiology^1^. Due to the limited availability of human islets, the vast majority of all physiological and pharmacological studies on the regulation of islet hormone secretion have been carried out using isolated rodent islets, particularly from a variety of mice strains. Although islets are readily available from both normal and transgenic mice, it is unclear to what extent data obtained by studying rodent islet function are translatable to the human context. In fact, mouse and human islets have distinct morphological arrangements, which may impact on their function^10^,^11^ and several studies comparing the gene expression profiles of human and rodent islets suggest that there are significant differences, including in GPCR expression^12^,^13^,^14^. This has been confirmed functionally in experiments using isolated rodent and human islets where it has been reported that human islets express β-adrenergic receptors that are coupled to elevation of insulin secretion, while β-adrenergic agonists have no effect on insulin release from rat islets^15^. In addition, MT1 receptors, which are activated by the pineal hormone melatonin, are reported to be expressed by rodent β-cells but not by human β-cells^16^, and different classes of purinergic receptor regulate intracellular calcium levels in rodent^17^ and human^18^ β-cells.

To date there has not been a systematic review of the GPCR gene expression repertoires of rodent and human islets, so in the current study we have compared human islet GPCR mRNA profiles with those of islets isolated from outbred ICR mice and inbred C57BL/6 mice. We have used these data to identify a core set of GPCRs that are expressed in both human and mouse islets, as well as islet GPCRs that show species-restricted expression. The extensive GPCR gene expression profiles presented here will provide the scientific community with a comprehensive comparative analysis of the mRNA expression of all functional human GPCRs and their mouse orthologues in human and mouse islets. This can then be used as a reference tool for the translation of mouse islet functional GPCR data to the human context, which will facilitate the evaluation of islet GPCRs as drug target candidates for novel diabetes therapies.

## Results

### Expression of GPCR mRNAs in islets isolated from C57 and ICR mice

**Figure 1:**
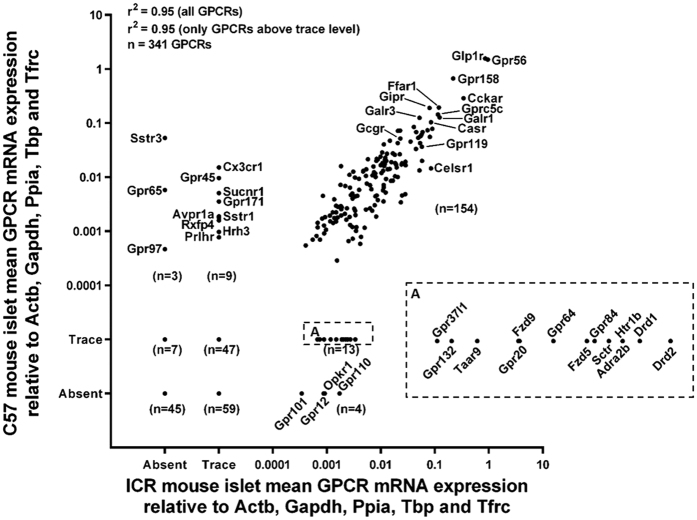
Comparison of the relative expression of 341 GPCR mRNAs in islets isolated from the outbred ICR and inbred C57 mouse strains. Insert panel A has been enlarged to allow visualisation of GPCRs expressed above trace level only in ICR mouse islets. Data are presented as mean GPCR expression relative to the reference genes Actb, Gapdh, Ppia, Tbp and Tfrc from non-pooled biological replicates from four C57 and four ICR mice.

The qPCR analyses indicated that transcripts encoding 183 GPCRs of the 341 GPCR mRNAs analysed (53.7%) were expressed at levels greater than 0.001% of the mean mRNA expression of the reference genes Actb, Gapdh, Ppia, Tbp and Tfrc in islets isolated from ICR and C57 mice. The remaining 158 GPCR mRNAs were either absent or expressed only at exceedingly low levels (<0.001% of the mean mRNA expression of the reference genes) in both mouse strains. It can be seen from Fig. 1 that the overall islet GPCR mRNA expression profiles in outbred ICR and inbred C57 mouse strains were very similar. Thus, although there were 52 fewer GPCRs expressed by C57 mouse islets (227 GPCR mRNAs vs 279 in C57 islets), there was a strong correlation between the expression patterns based on their levels relative to the reference genes (r^2^ = 0.95).

### Strain-specific expression of GPCR mRNAs in mouse islets

**Figure 2:**
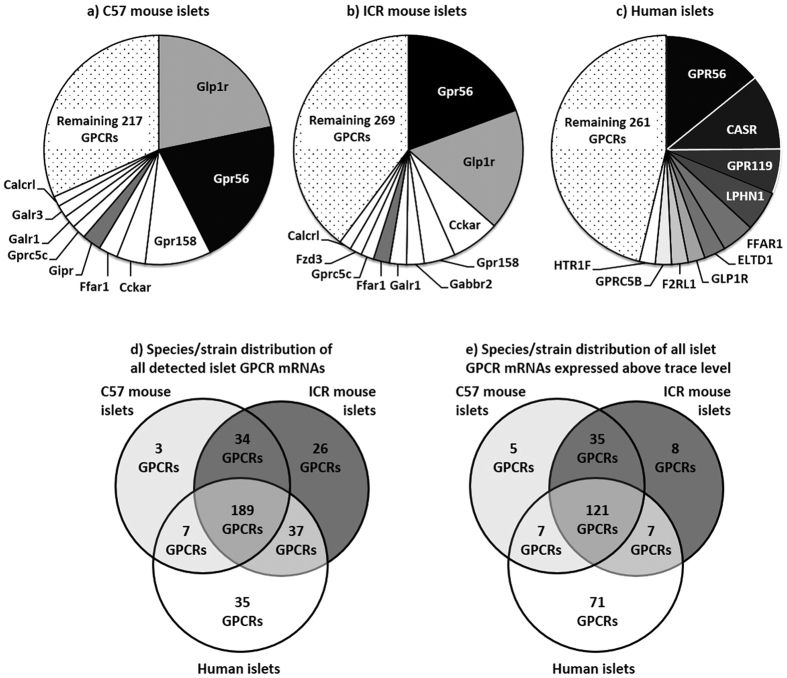
Summary of the relative expression of the ten most abundant GPCR mRNAs in outbred ICR mouse islets (**a**), inbred C57 mouse islets (**b**) and human islets (**c**). Colour coding in mouse islets according to similarities with human islet GPCR expression. White fields represent mouse islet GPCR mRNAs that are not among the ten most abundant GPCR mRNAs in human islets. Data for each GPCR are presented as mean % of the mRNA expression of all GPCRs in each islet type. (**d,e**) Species/strain distribution of all islet GPCR mRNAs in C57 and ICR mouse islets and human islets with all GPCR mRNAs (**d**) and only with GPCR mRNAs expressed above trace level (**e**).

154 of the 183 GPCR mRNAs that were detected above trace levels were commonly expressed by both ICR and C57 islets, indicating that there are striking similarities in islet GPCR expression between the two mouse strains. In fact, ICR and C57 mouse islets shared eight out of ten of their most abundantly expressed GPCR mRNAs (Calcrl, Cckar, Ffar1, Galr1, Glp1r, Gpr158, Gpr56 and Gprc5c; Fig. 2a,b), suggesting that these GPCRs play important roles in the normal function of mouse islets. However, we did identify some strain-specific differences in GPCR mRNA expression such that 12 GPCR mRNAs were expressed only by C57 islets while 17 additional GPCR mRNAs were only detected in ICR islets (Supplementary Table 1).

### Comparative analysis of GPCR mRNA expression by human and mouse islets

The relative expression of the ten most abundant GPCR mRNAs in human and mouse islets are presented in Fig. 2a–c. These figures indicate that while there was a high level of concordance between the mouse strains, as described above, only three of the ten most highly expressed human islet GPCR mRNAs were common to the ten most abundant mouse islet GPCR mRNAs. Nonetheless, in both mouse and human islets, the adhesion receptor GPR56 was the most abundantly expressed GPCR mRNA, with mRNA levels at ~20% and 50% of the mean mRNA expression of the five reference genes in human islets and mouse islets respectively. The GLP-1 receptor, which is the target for incretin mimetic therapies for type 2 diabetes that are known to stimulate insulin secretion in rodents and humans^19^, was another abundant GPCR in human and mouse islets. The third highly expressed GPCR mRNA common to human and mouse islets was FFAR1, which is activated by long chain fatty acids. The receptors identified in Fig. 2a–c were all expressed at >5% of the mean mRNA expression levels of the five reference genes and represented the most abundant GPCRs. However, overall the mean mRNA expression of all quantifiable islet GPCRs, relative to the reference genes, was 0.53% in human islets (206 GPCRs) and 1.4% in mouse islets (1.34% in ICR mouse islets; 167 GPCRs and 1.37% in C57 islets; 163 GPCRs). Thus, although there are a large number of GPCR mRNAs expressed by mouse and human islets, the majority of them are of low abundance. Expression of all human GPCR mRNAs and their mouse orthologues relative to ACTB, GAPDH, PPIA, TBP and TFRC are presented in Supplementary Figure 1.

The similarities and differences in islet GPCR mRNA expression between human islets and the two mouse strains are illustrated in Fig. 2d,e. The analysis was carried out with all detected GPCRs, including GPCRs present only at trace level (Fig. 2d) and also with only those GPCRs with mRNA expression above trace level (Fig. 2e), as these are more likely to affect islet function. A core set of 189 GPCR mRNAs (53.7% of all human GPCRs) were expressed in both human and ICR/C57 mouse islets (Fig. 2d). 9.9% of all GPCR mRNAs (35 GPCRs) were found exclusively in human islets, and 63 receptors (18.5% of all investigated GPCRs) were expressed exclusively in mouse islets and not in human islets (Fig. 2d). When only GPCR mRNAs present above trace level were examined, a core set of 121 GPCR mRNAs (34.4% of all GPCRs) was expressed in both human and ICR/C57 mouse islets. 20.7% of all GPCRs (71 receptors) were expressed above trace level only in human islets, whereas 48 receptors (14.1%) were expressed above trace level only in mouse islets (Fig. 2e).

**Figure 3:**
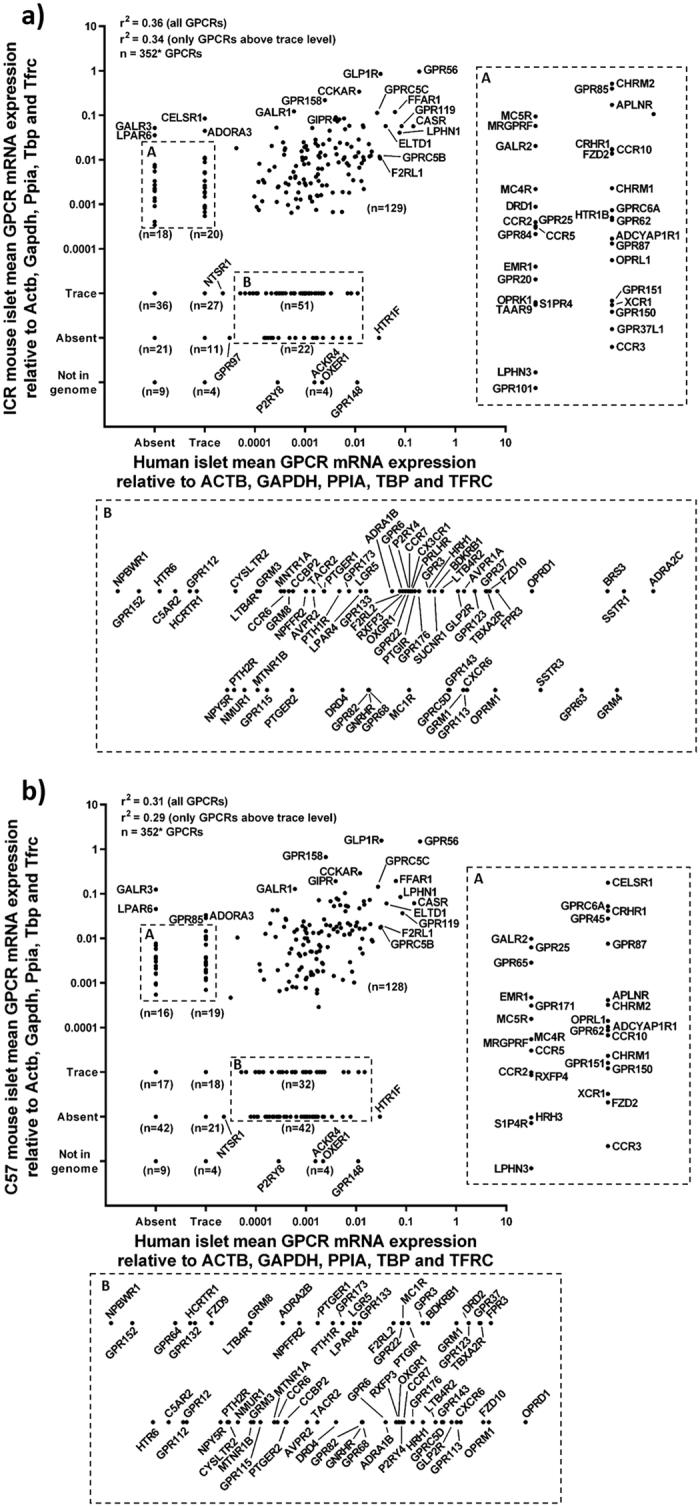
(**a,b**) Mean relative mRNA expression of human GPCRs in human islets and mouse GPCR orthologues in islets from ICR (**a**) and C57 (**b**) mice. Data are presented as mean GPCR mRNA expression ± SEM relative to the reference genes ACTB, GAPDH, PPIA, TBP and TFRC. Data generated using non-pooled islet RNA from four ICR and four C57 mice and four non-pooled human islet preparations. Insert panels A and B represent enlarged areas of the scatter plots, added to allow visualisation of individual GPCR mRNAs. *: excluding bitter taste receptors (TAS2Rs).

**Figure 4:**
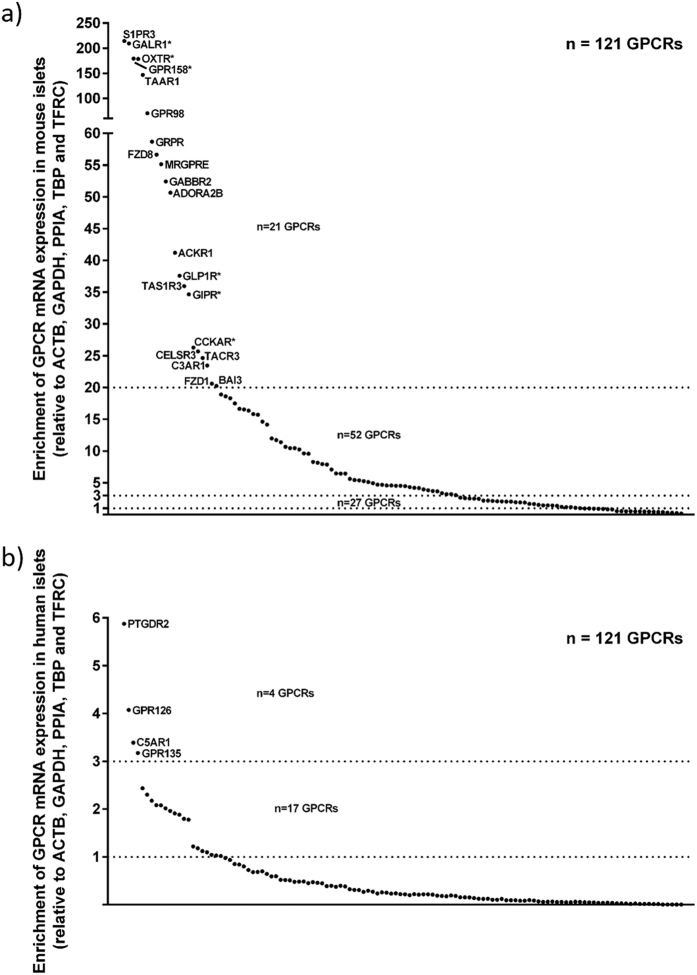
(**a,b**) Species enrichment of the 121 islet GPCR mRNAs expressed above trace level in both human and mouse islets. Mean ICR and C57 GPCR mRNA expression levels were compared to the mRNA expression of human islet GPCRs. mRNAs encoding 68 GPCRs were >3 fold enriched in mouse islets (**a**), 17 of these GPCRs (Adra2a, CalCrl, Cckar, Chrm3, Fzd3, Galr1, Gipr, Glp1r, Gpr116, Gpr125, Gpr158, Gpr56, Gprc5c, Lgr4, Oxtr, Ptger3, Vipr1) are also among the 25 most abundant mouse islet GPCR mRNAs (highlighted with * in figure a). Four GPCR mRNAs were >3 fold enriched in human islets (**b**). Data are presented as a ratio of the mean GPCR expression relative to the reference genes ACTB, GAPDH, PPIA, TBP and TFRC in human and mouse islets (i.e. mean of GPCR expression in ICR and C57 islets), and was generated using non-pooled islet RNA from four ICR and four C57 mice and four non-pooled human islet preparations.

There was a reasonable similarity in the GPCR mRNA expression profiles of human and mouse islets (ICR, r^2^: 0.36; C57, r^2^: 0.31), but the inter-species differences were much greater than the small inter-strain differences, as expected. Our data indicate that ~36% of all GPCRs screened were expressed above trace levels in both human and mouse islets, and a core set of 121 GPCR mRNAs were expressed by islets of both species (Fig. 3a,b). The enrichment of mRNAs encoding these 121 GPCRs in human and mouse islets was determined relative to mRNA expression levels of the five reference genes to allow direct comparison of GPCR expression patterns between species (Fig. 4a,b). This indicated that although a large number of GPCR mRNAs are expressed by both human and mouse islets, there are significant species differences in their abundance. Compared to human islets, mRNAs encoding 68 GPCRs were at least 3 fold enriched in mouse islets, and 17 of these GPCRs (Adra2a, CalCrl, Cckar, Chrm3, Fzd3, Galr1, Gipr, Glp1r, Gpr116, Gpr125, Gpr158, Gpr56, Gprc5c, Lgr4, Oxtr, Ptger3, Vipr1) were also among the 25 most abundant mouse islet GPCR mRNAs (Fig. 3a,b). Four GPCR mRNAs (GPR44, GPR126, C5AR1, GPR135) were >3 fold enriched in human islets compared to mouse islets (Fig. 3a,b). Overall, 72 of the 121 islet GPCR mRNAs expressed by both human and mouse islets differed <3 fold in mRNA expression.

**Figure 5:**
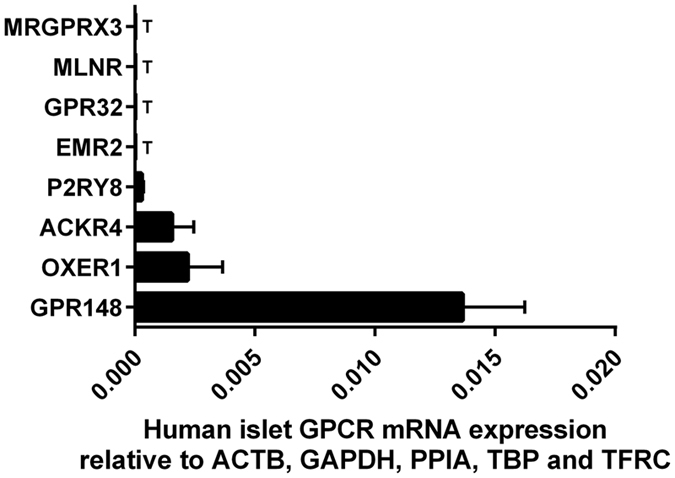
Relative expression in human islets of GPCRs that are absent in the mouse genome. Data are presented as mean ± SEM GPCR expression relative to the reference genes ACTB, GAPDH, PPIA, TBP and TFRC. n = four human islet donors. T denotes trace expression.

Approximately 10% of all GPCR mRNAs (ICR: 38 GPCRs; C57: 35 GPCRs; 43 GPCRs in total) were expressed exclusively in mouse islets, with either trace or no expression at all in human islets. In particular, mRNAs encoding the GAL_1_ (GALR1), GAL_2_ (GALR2) and GAL_3_ (GALR3), CELSR1, adenosine A_3_ (ADORA3), LPA_6_ (LPAR6) and GPR85 receptors were abundantly expressed in mouse islets but were either absent or expressed at very low level in human islets (Supplementary Figure 1). Similarly, we found that 81 GPCR mRNAs were preferentially expressed in human islets and were absent or expressed only at trace level in islets isolated from one or both of the mouse strains. The most abundant of the human islet-specific GPCR mRNAs were those encoding the 5-HT_1F_ (HTR1F), secretin (SCTR), α_2C_ adrenergic (ADRA2C), sst_1_ (SSTR1) and mGlu_4_ (GRM4) receptors (Supplementary Figure 1). In addition, three human-specific GPCR genes (GPR148, OXER1 and P2RY8), which do not exist in the mouse genome, were also expressed above trace levels in human islets (Fig. 5).

### Functional validation of human or mouse islet-specific GPCR expression

**Figure 6:**
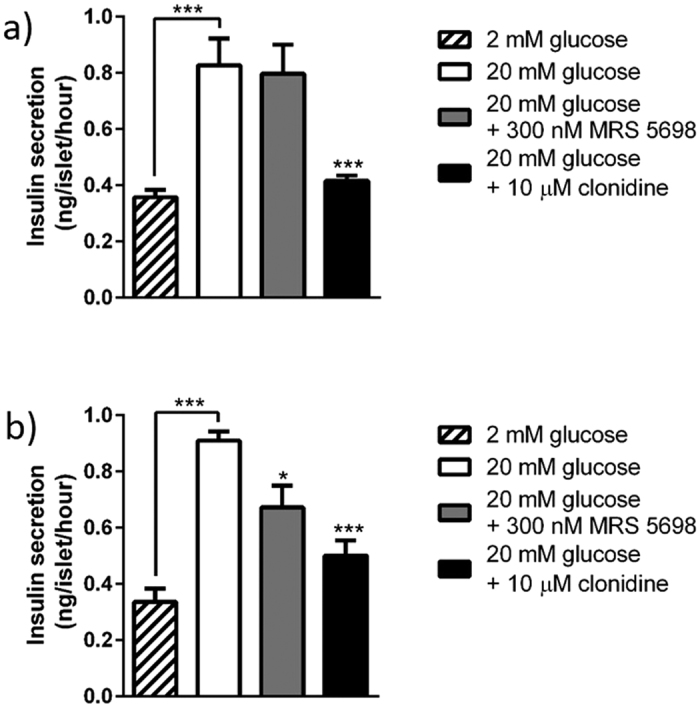
Confirmation of mouse islet-specific expression and function of the A_3_ (Adora3) receptor. The A3 receptor agonist MRS 5698 had no effect on insulin secretion from human islets (**a**), but significantly inhibited glucose-stimulated insulin secretion from mouse islets (**b**). The G_i_-coupled control agonist clonidine had significant inhibitory effects in both mouse and human islets. *p < 0.05; ***p < 0.001 versus secretion at 20 mM glucose.

**Figure 7:**
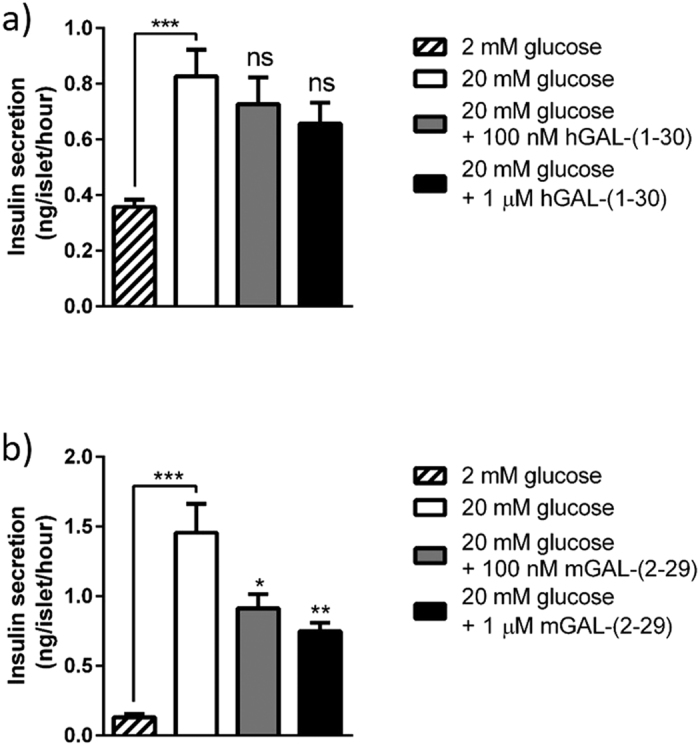
The effect on insulin secretion by the galanin receptor agonist galanin in human (**a**) and mouse (**b**) islets. Human galanin-(1–30) had no effect on insulin secretion from human islets, whereas insulin secretion from mouse islets was significantly inhibited by mouse galanin-(2–29) (**b**). n = 8 islets in each treatment group. *p < 0.05; **p < 0.01; ***p < 0.001.

**Figure 8:**
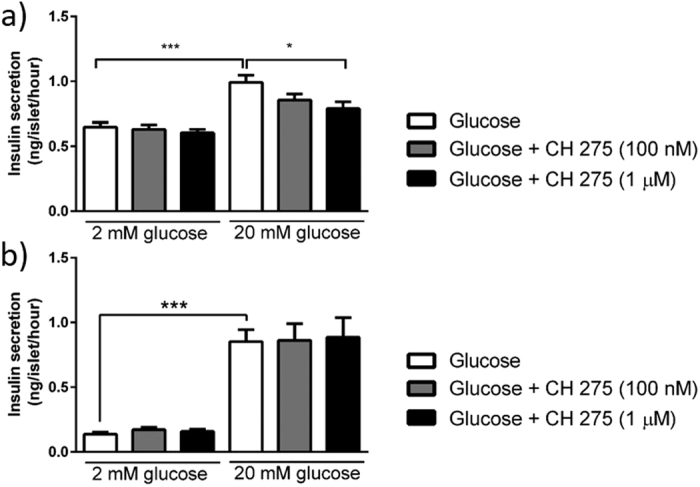
The effect of the Sst1 receptor agonist CH 275 on the secretion of insulin from human (**a**) and mouse (**b**) islets at 2 and 20 mM glucose. A dose-dependent inhibition of insulin secretion by CH 275 was observed in human islets (**a**), whereas CH275 had no effect on insulin secretion from mouse islets (**b**). *p < 0.05; ***p < 0.001.

Selective expression of A_3_ receptors by mouse islets was confirmed by experiments using the A_3_ receptor agonist MRS 5698, which caused significant inhibition of glucose-induced insulin secretion from mouse, but not human islets. Appropriate G_i_-coupling in human islets was confirmed by the use of the α_2_-adrenergic-selective agonist clonidine, which inhibited insulin secretion, as expected (Fig. 6b,c). Similarly, activation of the highly expressed galanin receptors on mouse islets by exposure to mouse galanin-(2–29) resulted in concentration-dependent inhibition of insulin secretion, whereas human galanin-(1–30) did not significantly affect insulin secretion from human islets (Fig. 7a,b). The G_i_-coupled sst1 (SSTR1) receptor mRNA was abundantly expressed in human islets, but only present at very low levels in mouse islets. Functional evaluation of the sst1 receptor agonist CH 275 indicated that it significantly inhibited insulin secretion from human islets, but it had no effect on insulin release from mouse islets (Fig. 8a,b).

## Discussion

A recent review analysing drugs acting on human therapeutic targets indicated that GPCRs are the largest class of targets for the development of new pharmaceutical products^20^. However, despite the use of GPCR-targeted drugs to treat a variety of clinical conditions such as asthma and hypertension only the GLP-1 receptor has been used successfully in treating T2D. This limited application of GPCR-based therapies for T2D is due, in part, to the poor functional characterisation of the majority of islet-expressed GPCRs and this is further exacerbated by the insufficient availability of human islets for identifying functional effects of GPCRs that are known to be expressed by islets. Rodent islets, in particular those isolated from mice, are therefore often relied upon as translational models. Structural differences between rodent and human islets are well documented^10^,^11^, which poses a question of the suitability of using rodent islets, in particular mouse islets, as translation models for predicting GPCR-mediated regulation of human islet function. Therefore, in this study, we have objectively evaluated the extent of the similarity of GPCR expression between the primary therapeutic target tissue, human islets, and the primary model system tissue, mouse islets.

Quantification of gene expression is the method of choice for systematic comparison of GPCR expression by isolated human and mouse islets, and qPCR is a highly sensitive, cost-effective and versatile method for detecting low abundance GPCRs^21^. Microarray has also traditionally been used for the quantification of gene expression, but this technology is less suitable for the detection of low abundance transcripts, which represent the majority of all GPCR mRNAs^22^. More recently, RNA-sequencing has emerged as a suitable alternative to qPCR. However this approach is still relatively costly, requires complex computational biology to fully analyse cellular transcriptome data and it requires post-analysis validation of gene expression data by a different platform, such as qPCR, which more closely mirrors gene expression data obtained by RNA-sequencing than by microarray^22^,^23^,^24^. Thus, the use of qPCR with validated primers for mRNAs encoding GPCRs, as used here, provides a focused strategy for defining and comparing the GPCRomes of mouse and human islets.

As expected, there was a very close correlation between GPCR mRNA profiles of islets isolated from the outbred ICR strain and inbred C57 strain of mice. Nonetheless, differences were observed, which may have an impact on the choice of model for functional characterisations. For example, C57 mouse islets did not express the sst3 receptor (Sstr3) so islets from this strain would not be suitable for characterising somatostatin signalling through this receptor subtype. Conversely, the orphan GPCR Gpr45 is expressed at moderately high levels in C57 islets but only at very low levels in ICR islets. This suggests that islets from C57 mice would be the model of choice for identifying the role of this receptor once appropriate tools are available, although relevance to the human situation is very low since GPR45 mRNA is only expressed at trace levels by human islets. In addition, although eight out of the ten most highly expressed GPCR mRNAs were common to C57 and ICR islets, Fzd3 and Gabbr2 were higher in ICR islets while Galr3 and Gipr were higher in C57 islets. Despite this, there was still a high similarity of expression levels of Fzd3, Gabbr2, Galr3 and Gipr between ICR and C57 islets, as shown in Supplementary Figure 1.

There have been earlier comparisons of gene expression in mouse and human islets^12^,^13^,^14^, but no focused study aimed at identifying similarities and differences in GPCR mRNA expression in human and mouse islets has been reported. Understanding which GPCRs are present on human islets, and if mouse islets can be used as a translational model system, are essential for evaluating whether particular islet GPCRs are potential novel targets for the development of new diabetes therapeutics. Interestingly, we have shown that GLP-1 receptors are in the top 10 GPCRs expressed by both mouse and human islets, so discoveries of the beneficial effects of GLP-1 agonists on glucose homeostasis in mice are directly translatable to the human situation, as is seen with the successful use of stable GLP-1 analogues as T2D therapies. We also found that FFAR1, which codes for a GPCR that is activated by long chain fatty acids, is highly expressed in human and mouse islets. FFAR1 agonists have been considered as novel therapies for T2D, but the development of a promising candidate, TAK-875^25^, has been discontinued because of adverse effects on the liver^26^.

We have also identified that several well-characterised GPCRs (CASR, GPRC5B, GPR119, F2RL1), which are either current drug targets or under evaluation as potential diabetes therapeutic targets, are highly expressed in both human and mouse islets. In addition, other highly expressed human islet GPCRs (e.g. ADGRG1 [GPR56], LPHN1, ELTD1) provide scope for further analysis, and the observation that ADGRG1 is also highly expressed in mouse islets indicates that these can be used with confidence to identify signalling downstream of this receptor. An important role for ADGRG1 in β-cell function and survival has recently been reported^27^, whereas the role of ELTD1 and LPHN1 in islet function remain unknown.

Our qPCR analyses have also indicated that a large number of GPCR mRNAs were expressed at very low levels (<0.001% of the mean mRNA expression of the five reference genes ACTB, GAPDH, PPIA, TBP and TFRC) in both human and mouse islets. At such low levels, it is not possible to accurately quantify the mRNA expression, as it falls outside of the linear range of detection for the QuantiTect qPCR primer assays. These mRNAs are nevertheless present in human and mouse islets, albeit at low copy numbers, and they are likely to be translated into GPCR proteins. It is difficult to evaluate the physiological impact of these low abundance receptor proteins on islet function. In fact, mRNAs encoding receptors present at trace levels are expressed at approximately 16,000–45,000 fold lower concentrations than the mRNA encoding the most abundant islet GPCR, ADGRG1 [GPR56]. We cannot exclude that GPCR mRNAs present at trace level may be transcribed into proteins capable of influencing islet function, but based on the assumption that the mRNAs encoding these receptors are expressed at very low levels, and therefore likely to result in low levels of GPCR protein, we hypothesise that GPCRs with mRNA expression at trace level in islets are less likely to have a major impact on islet function compared to GPCRs with more abundant mRNA expression. This is exemplified by our functional studies on the inhibitory effects of the A_3_ and galanin receptors on insulin secretion: mRNAs encoding the A_3_, GAL_1_ and GAL_3_ receptor mRNAs are highly expressed in mouse islets, but absent or only present at trace levels in human islets, and this in turn translated into a clear inhibitory effect on insulin secretion following activation of the GAL_1_/GAL_3_ or A_3_ receptors by exogenous agonists in mouse islets, while there was no significant effect on insulin secretion from human islets. Similar species-specific functional effects were observed for activation of the sst1 receptor, which is abundantly expressed in human islets but only present at very low levels in mouse islets. Consequently, in human islets, activation of the G_i_-coupled sst_1_ receptor by the selective sst1 receptor agonist CH 275 led to inhibition of insulin secretion, whereas no effects on mouse islet insulin secretion were observed.

The importance of determining the expression by human islets of GPCRs that have been investigated in rodent studies is illustrated by earlier studies where there is a mismatch between GPCR expression by mouse and human islets. For example, it has been reported that mRNA expression of the trace amine receptor Taar1 is restricted to islets in mice^8^, suggesting that this receptor might be a potential target for novel diabetes therapeutics. However, subsequent studies by ourselves and others using both qPCR and RNA-sequencing have shown that TAAR1 is expressed at very low levels in human islets^1^,^28^, thus making it a much less likely target for islet GPCR-focused therapeutics. Similarly, the abundant expression of several other mouse islet GPCRs - A_3_ (Adora3), Celsr1, GAL_1_ (Galr1), GAL_2_ (Galr2), GAL_3_ (Galr3), Gpr158, Lpar6, S1pr3 and OT (Oxtr) - can also be ruled out as potential human islet GPCR drug targets based on their low (or absent) expression in human islets.

In summary, in this study, we have systematically compared the expression of all known human GPCR mRNAs and their mouse orthologues in human and mouse islets and mapped the similarities and differences in islet GPCR mRNA expression between the two species. We have demonstrated that there is a common expression of a number of islet GPCRs between mouse and man, which allows mouse islets to be used as appropriate surrogates for human islets when investigating the function of these GPCRs in islets. However, we have also identified that significant differences do exist in GPCR expression between mouse and human islets, so care should be taken in the choice of model for functional analyses to ensure that data obtained using mouse islets are relevant to the human situation. Overall, the data presented here provide an essential resource for the translation of mouse islet functional data to the human islet context, which will facilitate the identification of islet GPCRs for evaluation as potential novel drug targets for diabetes.

## Research Design and Methods

### Islet isolation and culture

Islets were isolated from 10–12 week old male mice of the inbred C57BL/6 (C57; Charles River) and outbred ICR (Harlan) strains by collagenase digestion of the exocrine pancreas^29^. All animal procedures were approved by the King’s College London Ethics Committee and carried out in accordance with the UK Home Office Animals (Scientific Procedures) Act 1986.

Human islets were isolated from non-diabetic organ donors at the King’s College London Human Islet Isolation Unit^30^ with appropriate ethical approval and donor consent for research (Protocol number 01–082, Human Islet Isolation and Research). An assent form was completed by a relative of the pancreas donor for all islets used for research, and all experiments carried out on human islets were performed in accordance with the relevant guidelines and regulations as set out by the King’s College London Ethics Committee.

Isolated mouse and human islets were maintained in culture overnight (mouse: RPMI 1640; human: CMRL) at 37 °C, 5% CO_2_ before extraction of RNA.

### RNA extraction and quantitative real-time PCR

Olfactory, pseudogene and vomeronasal GPCRs were not included in the study and the bitter taste receptors (TAS2Rs) were also excluded as the nomenclature identifying the exact mouse TAS2R orthologues is very unclear, often with identical gene symbols being used for several different mouse bitter taste receptor genes. Total non-pooled RNA from ~200 islets isolated from four C57 and four ICR mice and from four non-diabetic human islet donors (2 male and 2 female, donor age 49.5 ± 3.5 years old, BMI 28.2 ± 2.3 kg/m^2^) was isolated using a modified TRIzol protocol followed by RNA clean-up on RNEasy MinElute columns (Qiagen)^31^. 500 ng of islet total RNA from each mouse or human donor was converted into cDNA using the TaqMan RT-PCR kit (Life Technologies) as described elsewhere^21^,^32^. Quantitative real-time PCR (qPCR) with Qiagen QuantiTect SYBR Green mouse or human primer assays was performed using islet cDNAs to quantify the expression of 352 non-olfactory, non-vomeronasal, non-bitter taste human GPCRs and 341 mouse orthologues as described in detail elsewhere^21^,^31^,^32^. For both mouse and human islets, GPCR mRNA levels were expressed relative to the reference genes ACTB, GAPDH, PPIA, TBP and TFRC. All GPCR and reference gene primer efficiency (E)^32^ values were in the range of 1.85–2.15. For all GPCR and reference gene quantifications, template cDNAs were diluted in such a way that all quantified genes returned Ct values <30. Genes expressed <0.001% of the mean mRNA level of the five reference genes were considered to be present only at trace level, as their expression was less than the lower limit of linear quantification of the QuantiTect primer assays. The human and mouse qPCR primers used for amplifications are listed in Supplementary Table 2. Enrichment of GPCR mRNA expression in human islets were calculated using the formula below:

*Enrichment of GPCR expression* = *Human islet GPCR expression relative to reference genes*/*Mean mouse islet GPCR expression relative to reference genes in ICR and C57 mouse islets*.

For enrichment of GPCR expression in mouse islets, the inverse of the above formula was used.

### Insulin secretion

Human and mouse isolated islets were cultured overnight in either CMRL or RPMI with 11 mM glucose supplemented with 10% fetal bovine serum. After a 1-h pre-incubation in physiological salt solution^33^ containing 2 mM glucose, insulin release was assessed by static incubation. Briefly, groups of five size-matched islets were hand-picked into 1.5 ml microcentrifuge tubes and incubated for 1 hour in a physiological salt solution at 37 °C in the absence or presence of the A_3_ receptor-selective agonist MRS 5698, human galanin-(1–30), mouse galanin-(2–29) or the sst1 agonist CH 275 (Tocris Bioscience). Insulin secreted into the supernatant was quantified by radioimmunoassay as described elsewhere^34^.

## Additional Information

**How to cite this article**: Amisten, S. *et al*. A comparative analysis of human and mouse islet G-protein coupled receptor expression. *Sci. Rep.* **7**, 46600; doi: 10.1038/srep46600 (2017).

**Publisher's note:** Springer Nature remains neutral with regard to jurisdictional claims in published maps and institutional affiliations.

## Supplementary Material

Supplementary Figures and Tables
