# Supplementary material for: A comparative analysis of human and mouse islet G-protein coupled receptor expression
Source: Sci Rep. 2017 Apr 19;7:46600. doi: 10.1038/srep46600 (PMC5395952; doi:10.1038/srep46600)
Supplement: Supplementary Figures and Tables [file srep46600-s1.pdf]

A comparative analysis of human and mouse islet G-protein coupled receptor expression

Stefan Amisten, Patricio Atanes, Ross Hawkes, Inmaculada Ruz-Maldonado, Bo Liu, Fariborz Parandeh, Min Zhao, Guo Cai Huang, Albert Salehi, Shanta J. Persaud

Supplementary figure 1. mRNA expression of human GPCRs and their mouse orthologues in human and mouse pancreatic islets relative to the reference genes ACTB, GAPDH, PPIA, TBP and TFRC. Data generated using four non-pooled human islet donors and four non-pooled ICR and four non-pooled C57 mouse islet preparations. T: trace mRNA expression; A: mRNA absent (i.e. not detected)

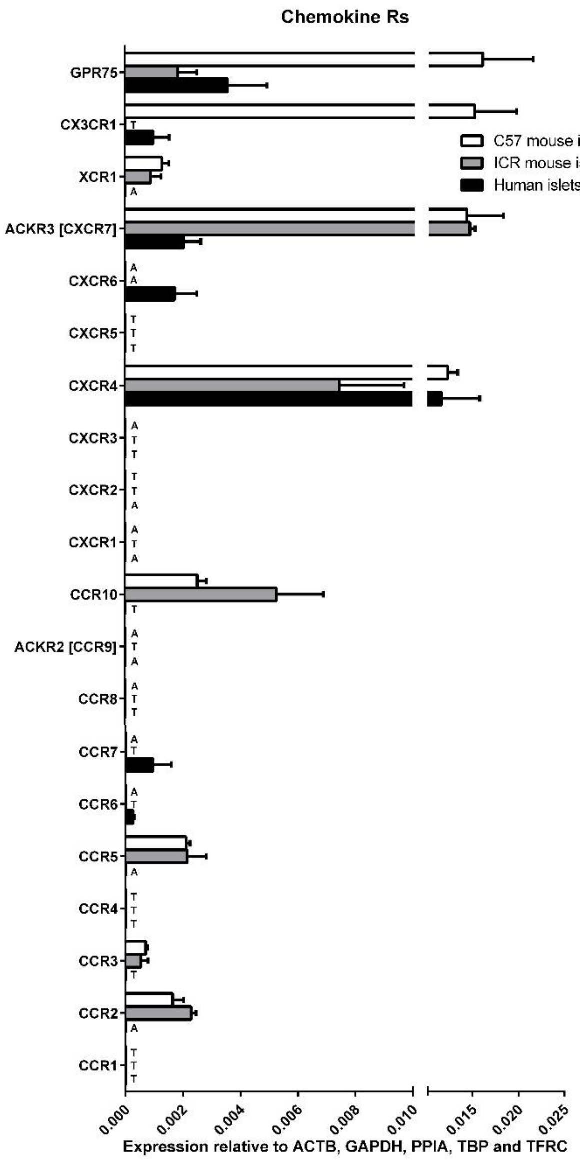
Chemokine Rs
GPR75
CX3CR1
XCR1
ACKR3 [CXCR7]
CXCR6
CXCR5
CXCR4
CXCR3
CXCR2
CXCR1
CCR10
ACKR2 [CCR9]
CCR8
CCR7
CCR6
CCR5
CCR4
CCR3
CCR2
CCR1
C57 mouse islets
ICR mouse islets
Human islets
0.000
0.002
0.004
0.006
0.008
0.010
0.015
0.020
0.025
Expression relative to ACTB, GAPDH, PPIA, TBP and TFRC

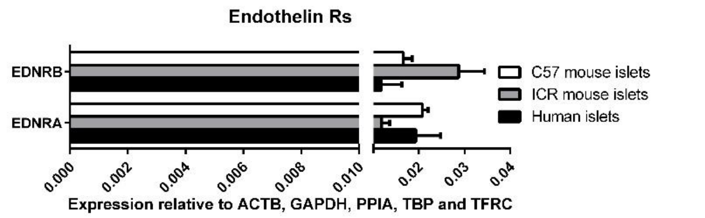
Endothelin Rs
EDNRB
EDNRA
C57 mouse islets
ICR mouse islets
Human islets
0.000
0.002
0.004
0.006
0.008
0.010
0.02
0.03
0.04
Expression relative to ACTB, GAPDH, PPIA, TBP and TFRC

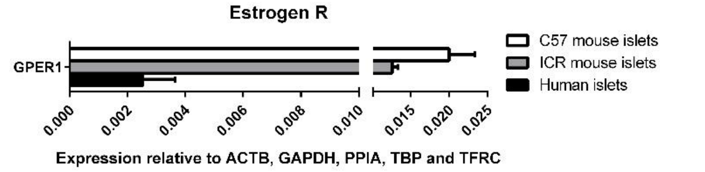
Estrogen R
GPER1
C57 mouse islets
ICR mouse islets
Human islets
0.000
0.002
0.004
0.006
0.008
0.010
0.015
0.020
0.025
Expression relative to ACTB, GAPDH, PPIA, TBP and TFRC

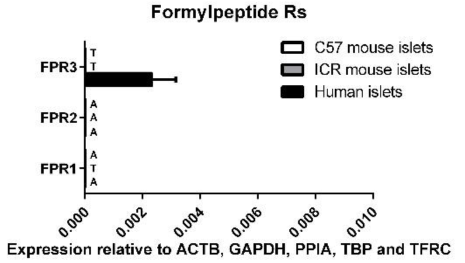
Formylpeptide Rs
FPR3
FPR2
FPR1
C57 mouse islets
ICR mouse islets
Human islets
0.000
0.002
0.004
0.006
0.008
0.010
Expression relative to ACTB, GAPDH, PPIA, TBP and TFRC

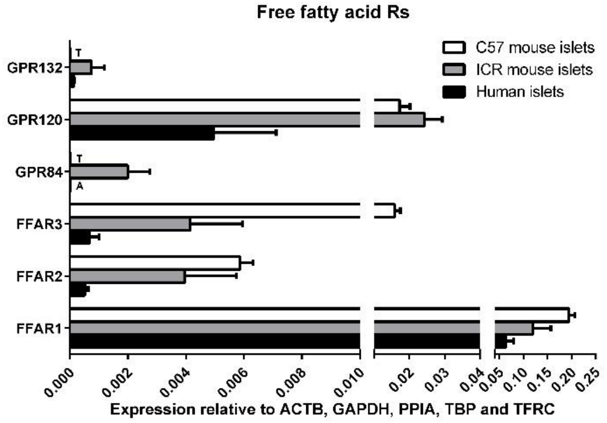
Free fatty acid Rs
GPR132
GPR120
GPR84
FFAR3
FFAR2
FFAR1
C57 mouse islets
ICR mouse islets
Human islets
0.000
0.002
0.004
0.006
0.008
0.010
0.02
0.03
0.04
0.05
0.10
0.15
0.20
0.25
Expression relative to ACTB, GAPDH, PPIA, TBP and TFRC

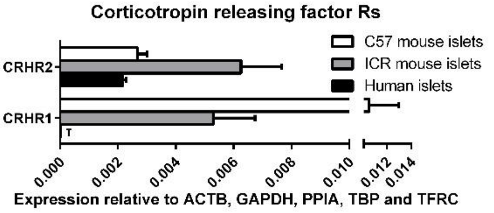
Corticotropin releasing factor Rs
CRHR2
CRHR1
C57 mouse islets
ICR mouse islets
Human islets
0.000
0.002
0.004
0.006
0.008
0.010
0.012
0.014
Expression relative to ACTB, GAPDH, PPIA, TBP and TFRC

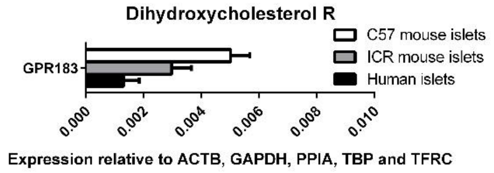
Dihydroxycholesterol R
GPR183
C57 mouse islets
ICR mouse islets
Human islets
0.000
0.002
0.004
0.006
0.008
0.010
Expression relative to ACTB, GAPDH, PPIA, TBP and TFRC

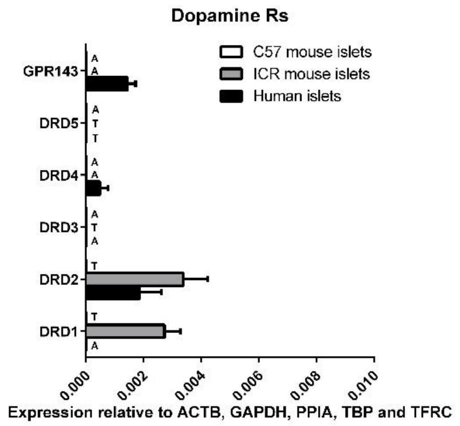
Dopamine Rs
GPR143
DRD5
DRD4
DRD3
DRD2
DRD1
C57 mouse islets
ICR mouse islets
Human islets
0.000
0.002
0.004
0.006
0.008
0.010
Expression relative to ACTB, GAPDH, PPIA, TBP and TFRC

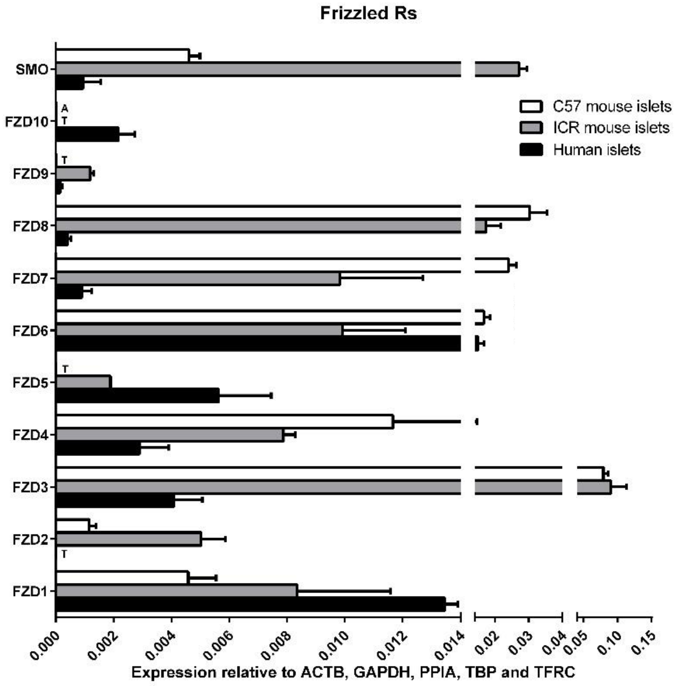
Frizzled Rs
SMO
FZD10
FZD9
FZD8
FZD7
FZD6
FZD5
FZD4
FZD3
FZD2
FZD1
C57 mouse islets
ICR mouse islets
Human islets
0.000
0.002
0.004
0.006
0.008
0.010
0.012
0.014
0.02
0.03
0.04
0.05
0.10
0.15
Expression relative to ACTB, GAPDH, PPIA, TBP and TFRC

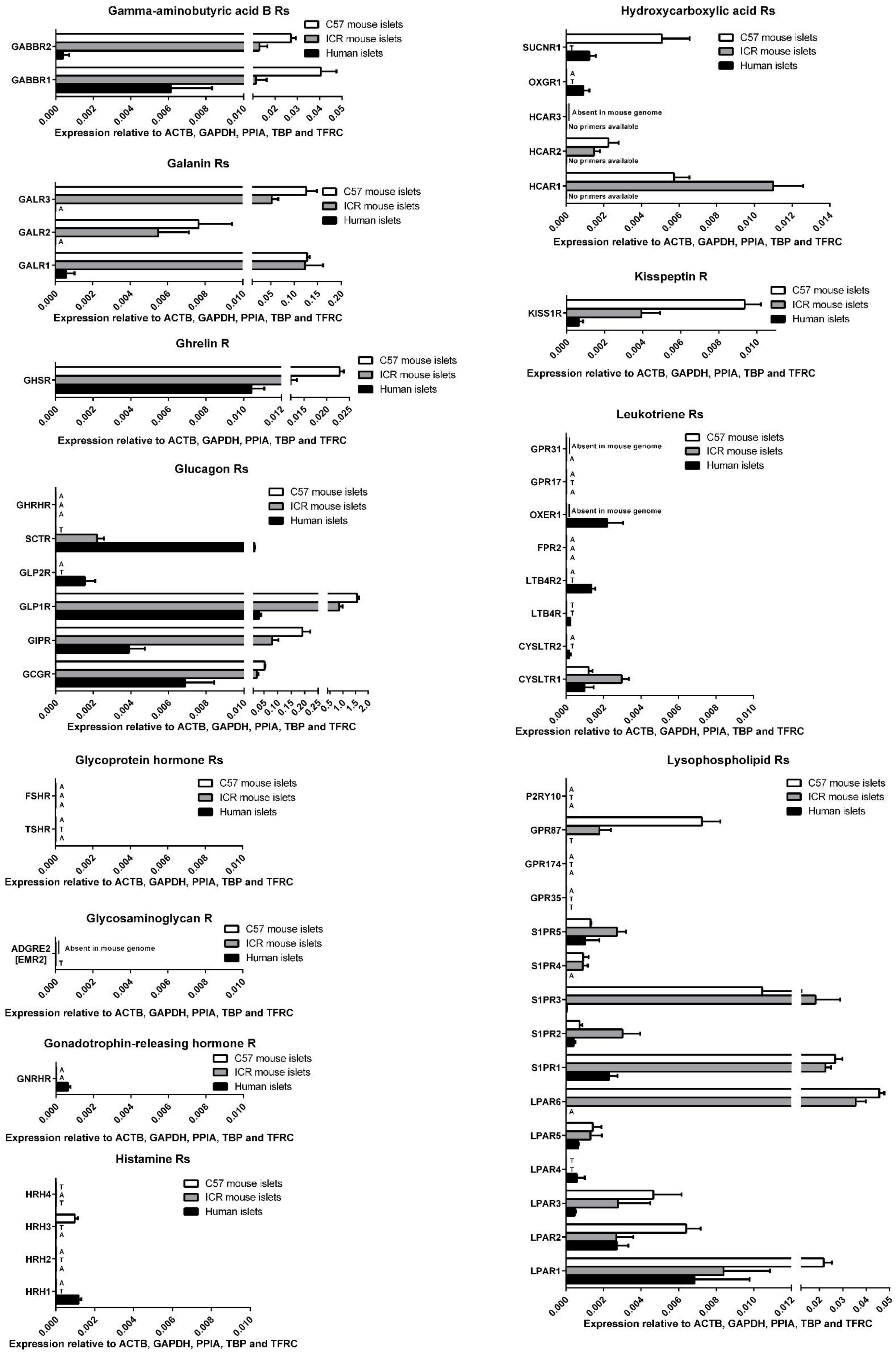

Gamma-aminobutyric acid B Rs
C57 mouse islets
ICR mouse islets
Human islets
GABBR2
GABBR1
0.000 0.002 0.004 0.006 0.008 0.010 0.02 0.03 0.04 0.05
Expression relative to ACTB, GAPDH, PPIA, TBP and TFRC
Galanin Rs
GALR3
GALR2
GALR1
0.000 0.002 0.004 0.006 0.008 0.010 0.05 0.10 0.15 0.20
Ghrelin R
GHSR
0.000 0.002 0.004 0.006 0.008 0.010 0.012 0.015 0.020 0.025
Glucagon Rs
GHRHR
SCTR
GLP2R
GLP1R
GIPR
GCGR
0.000 0.002 0.004 0.006 0.008 0.010 0.05 0.10 0.15 0.20 0.25 0.5 1.0 1.5 2.0
Glycoprotein hormone Rs
FSHR
TSHR
Glycosaminoglycan R
ADGRE2 [EMR2]
Absent in mouse genome
Gonadotrophin-releasing hormone R
GNRHR
Histamine Rs
HRH4
HRH3
HRH2
HRH1
Hydroxycarboxylic acid Rs
SUCNR1
OXGR1
HCAR3
HCAR2
HCAR1
Absent in mouse genome
No primers available
0.000 0.002 0.004 0.006 0.008 0.010 0.012 0.014
Kisspeptin R
KISS1R
Leukotriene Rs
GPR31
GPR17
OXER1
FPR2
LTB4R2
LTB4R
CYSLTR2
CYSLTR1
Lysophospholipid Rs
P2RY10
GPR87
GPR174
GPR35
S1PR5
S1PR4
S1PR3
S1PR2
S1PR1
LPAR6
LPAR5
LPAR4
LPAR3
LPAR2
LPAR1
0.000 0.002 0.004 0.006 0.008 0.010 0.012 0.02 0.03 0.04 0.05

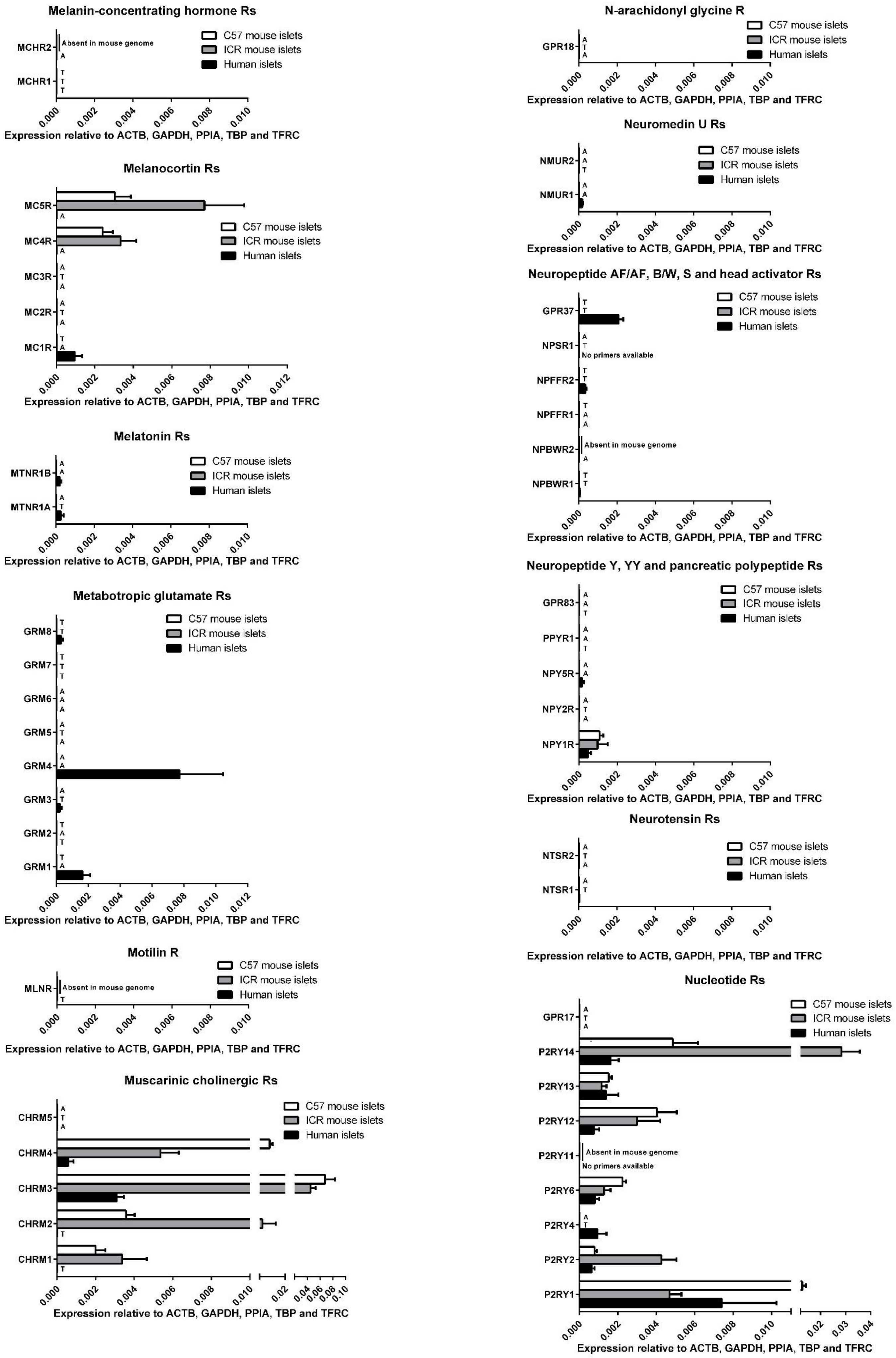
Melanin-concentrating hormone Rs
MCHR2
Absent in mouse genome
MCHR1
C57 mouse islets
ICR mouse islets
Human islets
Expression relative to ACTB, GAPDH, PPIA, TBP and TFRC
Melanocortin Rs
MC5R
MC4R
MC3R
MC2R
MC1R
Melatonin Rs
MTNR1B
MTNR1A
Metabotropic glutamate Rs
GRM8
GRM7
GRM6
GRM5
GRM4
GRM3
GRM2
GRM1
Motilin R
MLNR
Absent in mouse genome
Muscarinic cholinergic Rs
CHRM5
CHRM4
CHRM3
CHRM2
CHRM1
N-arachidonyl glycine R
GPR18
Neuromedin U Rs
NMUR2
NMUR1
Neuropeptide AF/AF, B/W, S and head activator Rs
GPR37
NPSR1
No primers available
NPFFR2
NPFFR1
NPBWR2
Absent in mouse genome
NPBWR1
Neuropeptide Y, YY and pancreatic polypeptide Rs
GPR83
PPYR1
NPY5R
NPY2R
NPY1R
Neurotensin Rs
NTSR2
NTSR1
Nucleotide Rs
GPR17
P2RY14
P2RY13
P2RY12
P2RY11
Absent in mouse genome
No primers available
P2RY6
P2RY4
P2RY2
P2RY1

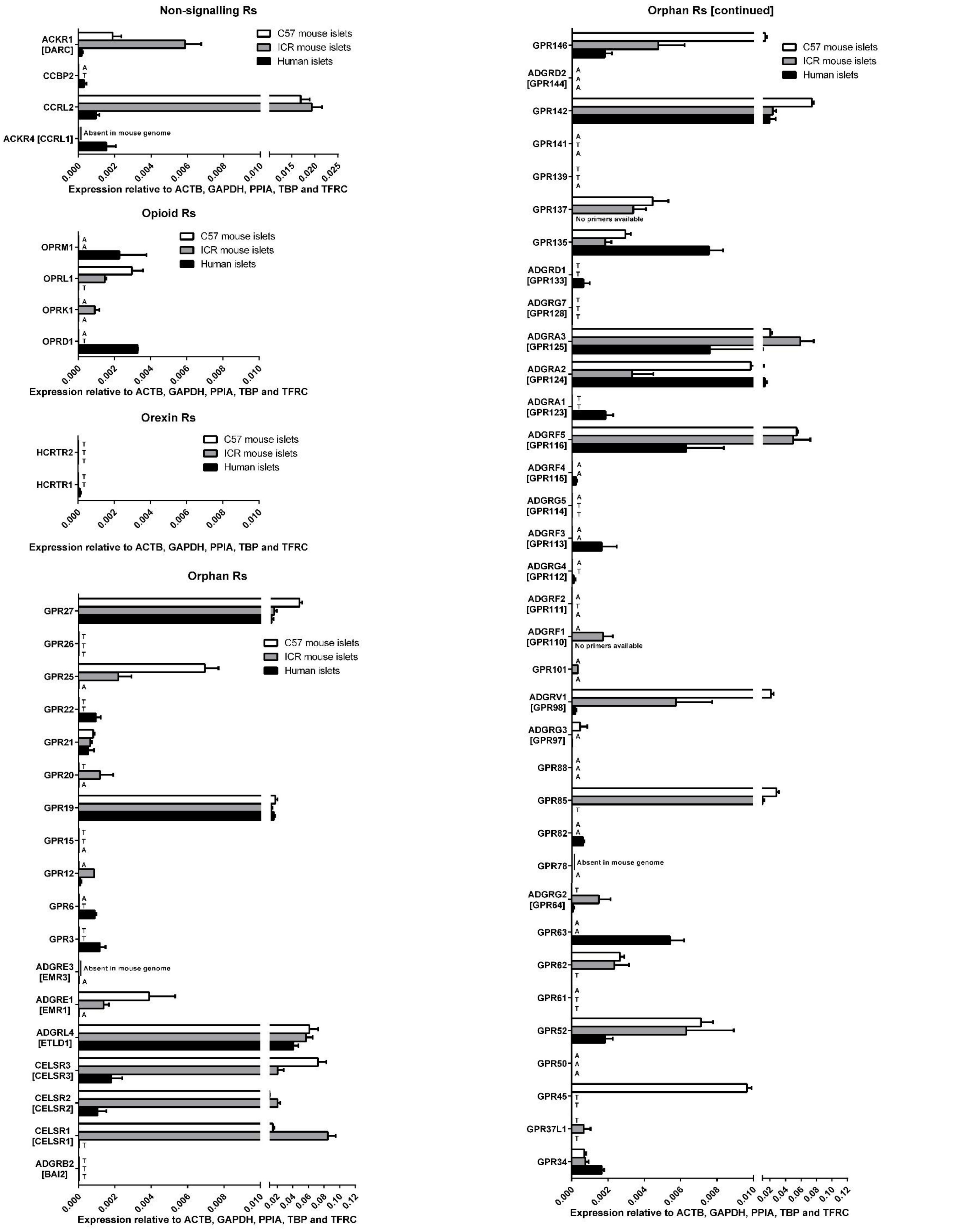
Non-signalling Rs
ACKR1 [DARC]
CCBP2
CCRL2
ACKR4 [CCRL1]
Absent in mouse genome
C57 mouse islets
ICR mouse islets
Human islets
Expression relative to ACTB, GAPDH, PPIA, TBP and TFRC
Opioid Rs
OPRM1
OPRL1
OPRK1
OPRD1
Expression relative to ACTB, GAPDH, PPIA, TBP and TFRC
Orexin Rs
HCRTR2
HCRTR1
Expression relative to ACTB, GAPDH, PPIA, TBP and TFRC
Orphan Rs
GPR27
GPR26
GPR25
GPR22
GPR21
GPR20
GPR19
GPR15
GPR12
GPR6
GPR3
ADGRE3 [EMR3]
Absent in mouse genome
ADGRE1 [EMR1]
ADGRL4 [ETLD1]
CELSR3 [CELSR3]
CELSR2 [CELSR2]
CELSR1 [CELSR1]
ADGRB2 [BAI2]
Expression relative to ACTB, GAPDH, PPIA, TBP and TFRC
Orphan Rs [continued]
GPR146
ADGRD2 [GPR144]
GPR142
GPR141
GPR139
GPR137
No primers available
GPR135
ADGRD1 [GPR133]
ADGRG7 [GPR128]
ADGRA3 [GPR125]
ADGRA2 [GPR124]
ADGRA1 [GPR123]
ADGRF5 [GPR116]
ADGRF4 [GPR115]
ADGRG5 [GPR114]
ADGRF3 [GPR113]
ADGRG4 [GPR112]
ADGRF2 [GPR111]
ADGRF1 [GPR110]
No primers available
GPR101
ADGRV1 [GPR98]
ADGRG3 [GPR97]
GPR88
GPR85
GPR82
GPR78
Absent in mouse genome
ADGRG2 [GPR64]
GPR63
GPR62
GPR61
GPR52
GPR50
GPR45
GPR37L1
GPR34
Expression relative to ACTB, GAPDH, PPIA, TBP and TFRC

Orphan Rs [continued]

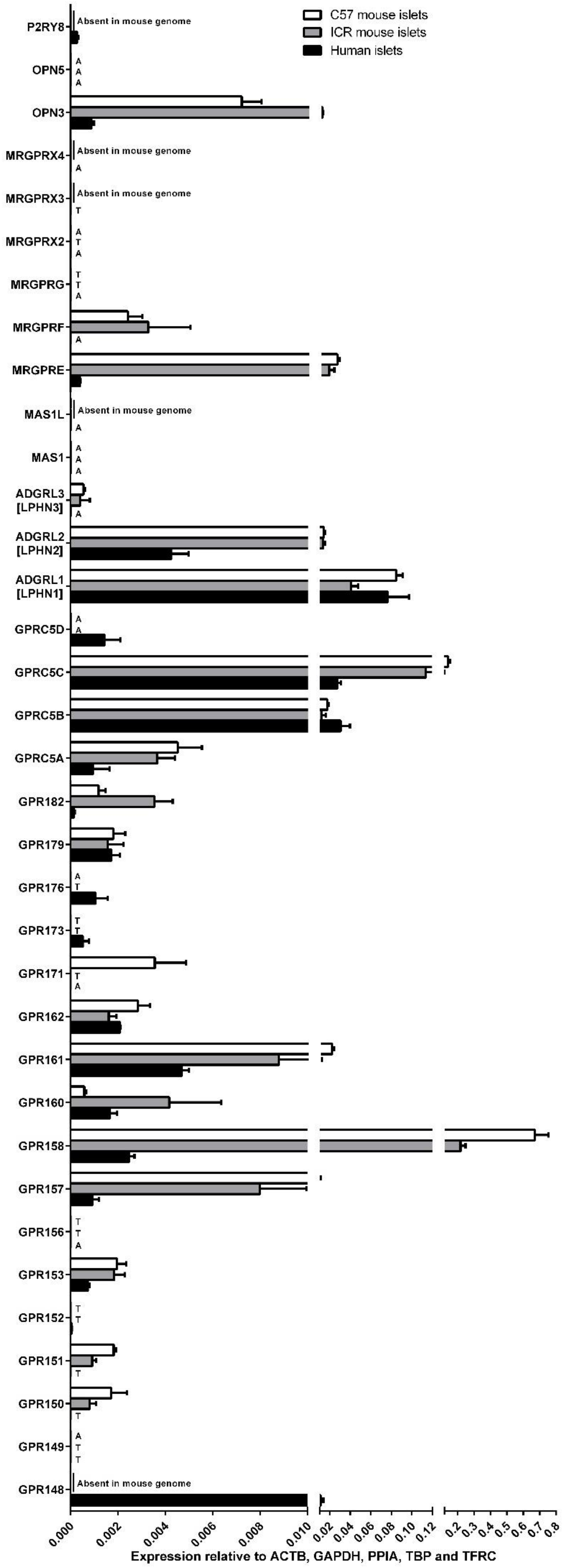

Parathyroid hormone Rs

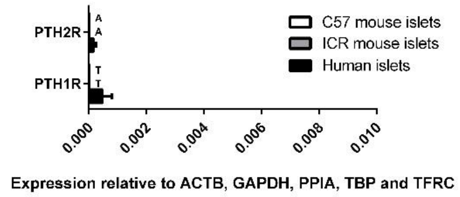

Peptide P518 R

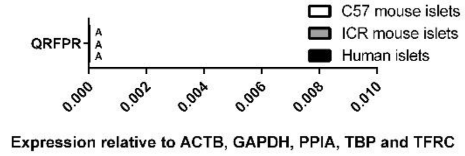

Platelet-activating factor R

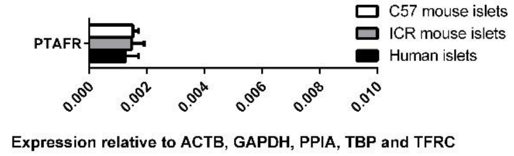

Prolactin-releasing peptide R

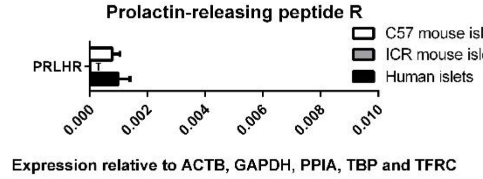

Prokineticin Rs

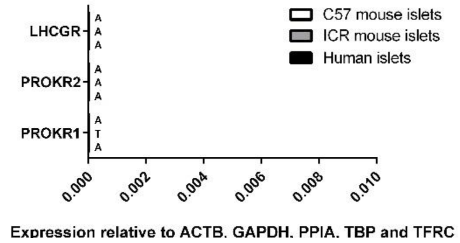

Prostanoid Rs

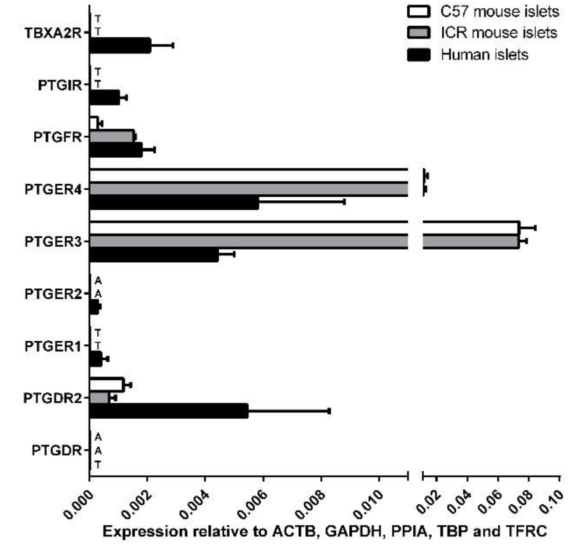

Protease-activated Rs

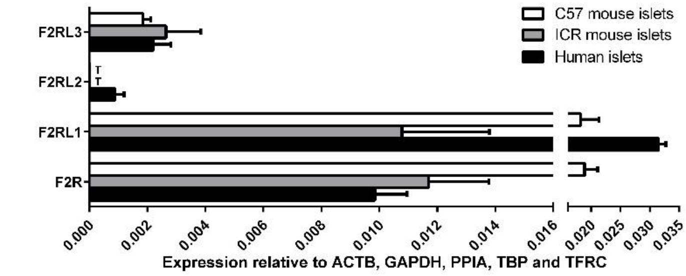

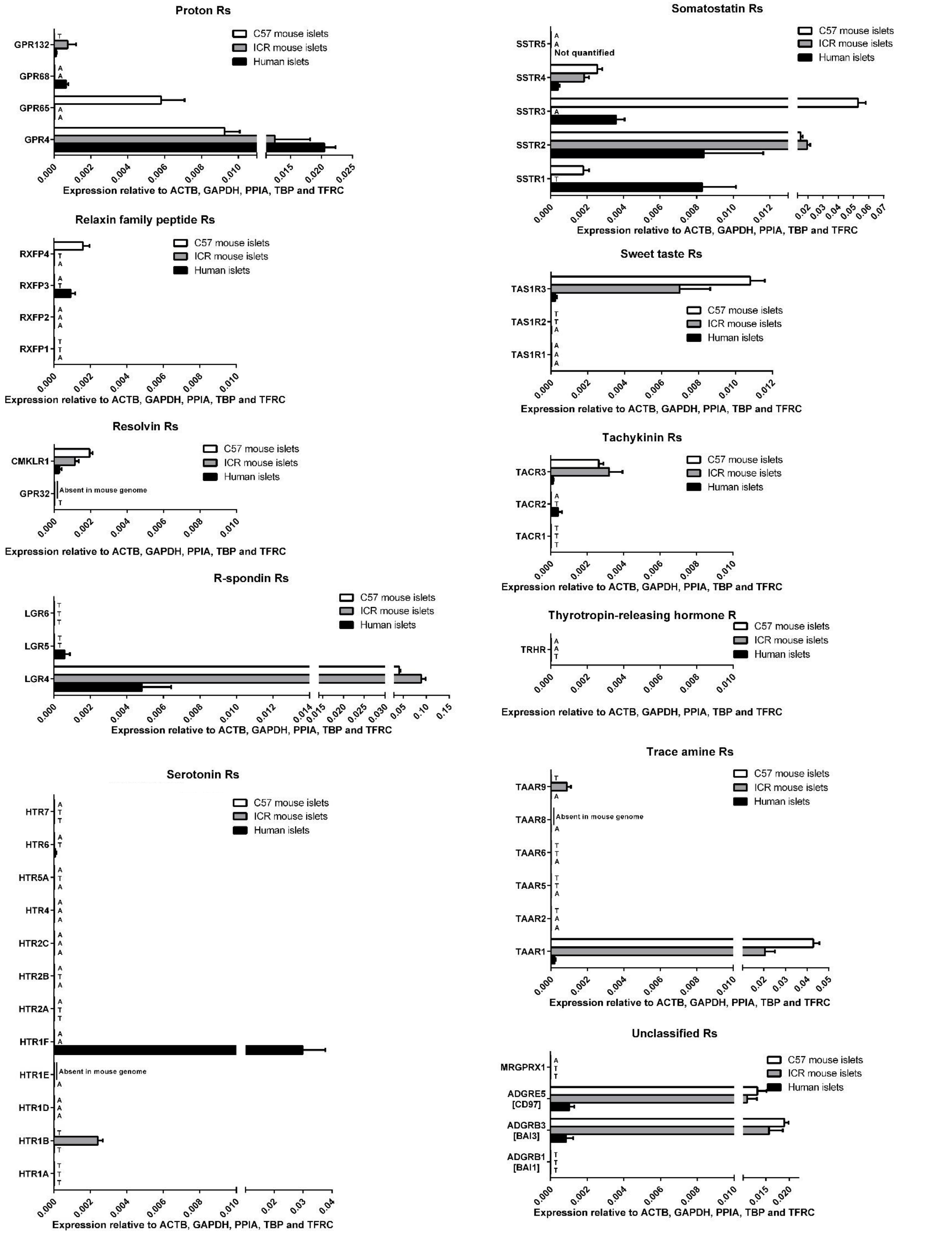

Proton Rs
GPR132
GPR68
GPR65
GPR4
C57 mouse islets
ICR mouse islets
Human islets
Expression relative to ACTB, GAPDH, PPIA, TBP and TFRC
Somatostatin Rs
SSTR5
Not quantified
SSTR4
SSTR3
SSTR2
SSTR1
Relaxin family peptide Rs
RXFP4
RXFP3
RXFP2
RXFP1
Sweet taste Rs
TAS1R3
TAS1R2
TAS1R1
Resolvin Rs
CMKLR1
GPR32
Absent in mouse genome
Tachykinin Rs
TACR3
TACR2
TACR1
R-spondin Rs
LGR6
LGR5
LGR4
Thyrotropin-releasing hormone R
TRHR
Serotonin Rs
HTR7
HTR6
HTR5A
HTR4
HTR2C
HTR2B
HTR2A
HTR1F
HTR1E
HTR1D
HTR1B
HTR1A
Trace amine Rs
TAAR9
TAAR8
TAAR6
TAAR5
TAAR2
TAAR1
Unclassified Rs
MRGPRX1
ADGRE5 [CD97]
ADGRB3 [BAI3]
ADGRB1 [BAI1]

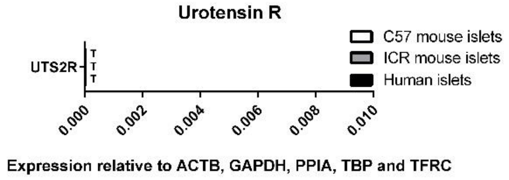
Urotensin R
C57 mouse islets
ICR mouse islets
Human islets
UTS2R
T
T
T
0.000
0.002
0.004
0.006
0.008
0.010
Expression relative to ACTB, GAPDH, PPIA, TBP and TFRC

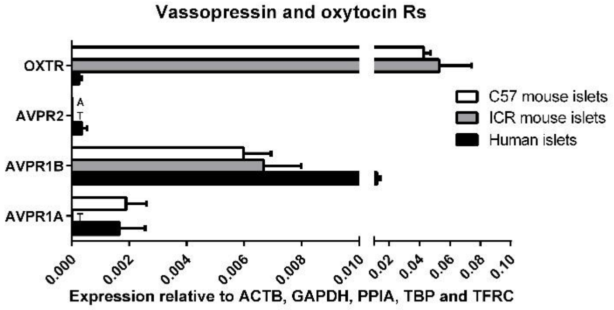
Vassopressin and oxytocin Rs
OXTR
AVPR2
A
T
AVPR1B
AVPR1A
T
C57 mouse islets
ICR mouse islets
Human islets
0.000
0.002
0.004
0.006
0.008
0.010
0.02
0.04
0.06
0.08
0.10
Expression relative to ACTB, GAPDH, PPIA, TBP and TFRC

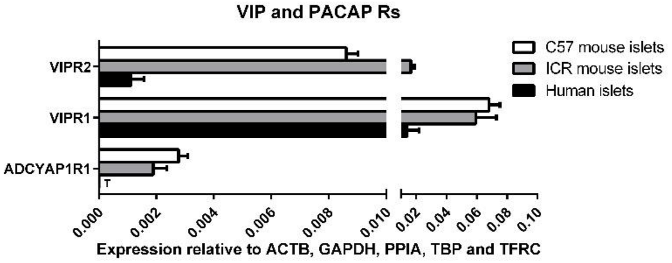
VIP and PACAP Rs
C57 mouse islets
ICR mouse islets
Human islets
VIPR2
VIPR1
ADCYAP1R1
T
0.000
0.002
0.004
0.006
0.008
0.010
0.02
0.04
0.06
0.08
0.10
Expression relative to ACTB, GAPDH, PPIA, TBP and TFRC

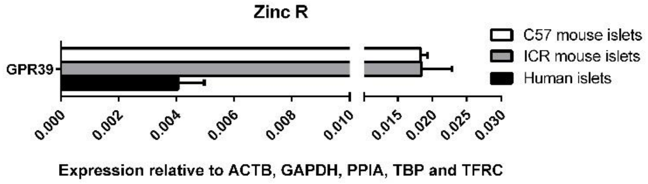
Zinc R
C57 mouse islets
ICR mouse islets
Human islets
GPR39
0.000
0.002
0.004
0.006
0.008
0.010
0.015
0.020
0.025
0.030
Expression relative to ACTB, GAPDH, PPIA, TBP and TFRC

Supplementary table 1. Differences in mouse islet GPCR mRNA expression between the C57 and ICR mouse strains. n=4 individual C57 or ICR mouse islet preparations.

Gene symbol	ICR mouse islet GPCR mRNA expression relative to 5 reference genes	C57 mouse islet GPCR mRNA expression relative to 5 reference genes
Adra2b	0.0024 ± 0.0013	trace
Drd1	0.0027 ± 0.0005	trace
Drd2	0.0034 ± 0.0009	trace
Fzd5	0.0019 ± 0.0000	trace
Fzd9	0.0012 ± 0.0001	trace
Gpr101	0.0003 ± 0.0000	absent
Gpr110	0.0017 ± 0.0005	absent
Gpr12	0.0009 ± 0.0000	absent
Gpr132	0.0007 ± 0.0004	trace
Gpr20	0.0012 ± 0.0007	trace
Gpr37l1	0.0007 ± 0.0004	trace
Gpr64	0.0015 ± 0.0006	trace
Gpr84	0.0020 ± 0.0008	trace
Htr1b	0.0024 ± 0.0003	trace
Oprk1	0.0009 ± 0.0003	absent
Sctr	0.0022 ± 0.0004	trace
Taar9	0.0009 ± 0.0002	trace
Avpr1a	trace	0.0151 ± 0.0045
Cx3cr1	trace	0.0035 ± 0.0013
Gpr171	trace	0.0096 ± 0.0003
Gpr45	trace	0.0058 ± 0.0013
Gpr65	absent	0.0005 ± 0.0004
Gpr97	absent	0.0010 ± 0.0002
Hrh3	trace	0.0008 ± 0.0003
Prlhr	trace	0.0016 ± 0.0004
Rxfp4	trace	0.0018 ± 0.0003
Sstr1	trace	0.0531 ± 0.0051
Sstr3	absent	0.0051 ± 0.0015
Sucnr1	trace	0.0151 ± 0.0045

Supplementary Table 2. Quantitative real-time PCR primers used to quantify human and mouse GPCRs and reference genes in human and mouse islets.

Gene symbol	Human primer assay	Mouse primer assay
ACKR1	QT00208719	QT01748663
ACKR2	QT00040432	QT00154105
ACKR3	QT00069650	QT00254443
ACKR4	QT01665951	QT01040753
ADCYAP1R1	QT00062181	QT00120561
ADORA1	QT01531635	QT00301119
ADORA2A	QT00210245	QT02527308
ADORA2B	QT00029911	QT00257558
ADORA3	QT00041181	QT01068347
ADRA1A	QT00010500	QT00129584
ADRA1B	QT00045458	QT01051876
ADRA1D	QT00199717	QT00147315
ADRA2A	QT00211967	QT00287063
ADRA2B	QT00211932	QT00311675
ADRA2C	QT00205765	QT01749328
ADRB1	QT00204309	QT00258692
ADRB2	QT00200011	QT00253967
ADRB3	QT00200004	QT01756160
AGTR1	QT00233548	QT00261464
AGTR2	QT00065758	QT00197540
APLNR	QT00221592	QT00254275
AVPR1A	QT00001344	QT00113169
AVPR1B	QT01848511	QT01753941
AVPR2	QT02290288	QT00315315
BAI1	QT02423575	QT00123228
BAI2	QT02423547	QT00158732
BAI3	QT00004473	QT01062873
BDKRB1	QT02423582	QT00326886
BDKRB2	QT01847209	QT00111027
BRS3	QT00020321	QT00250222
C3AR1	QT01676941	QT02380581
C5AR1	QT00997766	QT01164723
CALCR	QT02423589	QT00108864
CALCRL	QT01677032	QT00128786
CASR	QT00055944	QT00134015
CCKAR	QT00000105	QT00099673
CCKBR	QT00000126	QT00128597
CCR1	QT00047740	QT00156058
CCR2	QT01679986	QT02522849

CCR3	QT02423596	QT00262822
CCR4	QT00998795	QT00250432
CCR5	QT01336601	QT00114569
CCR6	QT01666140	QT02379181
CCR7	QT01666686	QT00240975
CCR8	QT02423834	QT01059184
CCR9	QT01670144	QT01165038
CCR10	QT00034783	QT00260673
CCRL2	QT00225988	QT01040179
CD97	QT01681498	QT00198919
CELSR1	QT00030681	QT01748096
CELSR2	QT00010948	QT01536724
CELSR3	QT00199437	QT00123865
CHRM1	QT00215404	QT01753311
CHRM2	QT01679398	QT00290297
CHRM3	QT01844346	QT01772169
CHRM4	QT00214963	QT00313712
CHRM5	QT01877155	QT00298452
CMKLR1	QT01680140	QT01060430
CNR1	QT02305702	QT02522457
CNR2	QT00012376	QT00159558
CRHR1	QT00059122	QT00106232
CRHR2	QT02423610	QT00151543
CX3CR1	QT00203434	QT00259126
CXCR1	QT00212919	QT01778434
CXCR2	QT00000518	QT00283696
CXCR3	QT02423617	QT00249438
CXCR4	QT00223188	QT00249305
CXCR5	QT02423624	QT00253449
CXCR6	QT01156918	QT02528806
CYSLTR1	QT00039368	QT01756503
CYSLTR2	QT00216902	QT00136885
DRD1	QT00200025	QT00263396
DRD2	QT00012558	QT01169063
DRD3	QT00020307	QT00170527
DRD4	QT00204316	QT00125489
DRD5	QT02423631	QT00281596
EDNRA	QT00030156	QT00121625
EDNRB	QT00014343	QT00139384
ELTD1	QT00025557	QT00170051
EMR1	QT00001519	QT00099617
EMR2	QT00075285	NA

EMR3	QT00041272	NA
F2R	QT00230489	QT00119812
F2RL1	QT00196966	QT02255330
F2RL2	QT00202139	QT00101178
F2RL3	QT02423456	QT00252168
FFAR1	QT01001567	QT00308833
FFAR2	QT01001574	QT00128226
FFAR3	QT01841952	QT00303499
FFAR4	QT00225925	QT00257033
FPR1	QT00199745	QT01165899
FPR2	QT00204295	QT00171514
FPR3	QT00054677	QT01063216
FSHR	QT02360260	QT00122472
FZD1	QT01157163	QT00290542
FZD2	QT02423638	QT00261485
FZD3	QT00009114	QT00147917
FZD4	QT00200984	QT00260526
FZD5	QT00200886	QT01063461
FZD6	QT00047670	QT00109998
FZD7	QT01010919	QT00307797
FZD8	QT00212128	QT02328151
FZD9	QT00241402	QT00248906
FZD10	QT00201782	QT00279979
GABBR1	QT00028539	QT01052541
GABBR2	QT00030345	QT01045912
GALR1	QT00041069	QT00108969
GALR2	QT00209552	QT00262836
GALR3	QT01011157	QT00326081
GCGR	QT00000133	QT00112560
GHRHR	QT00000427	QT01062243
GHSR	QT02402302	QT00138439
GIPR	QT00033138	QT01564913
GLP1R	QT00066780	QT00130767
GLP2R	QT00047320	QT01744197
GNRHR	QT02423841	QT00107205
GPBAR1	QT00209594	QT01038471
GPER1	QT00201040	QT00260659
GPR1	QT00209748	QT00124341
GPR3	QT00203014	QT00249732
GPR4	QT00200865	QT00264243
GPR6	QT00202272	QT00296044
GPR12	QT01160334	QT01062656

GPR15	QT01001497	QT00283717
GPR17	QT00009828	QT01043707
GPR18	QT01001504	QT00129227
GPR19	QT00203749	QT02242415
GPR20	QT00211288	QT00266735
GPR21	QT01001511	QT00322035
GPR22	QT00221977	QT00131775
GPR25	QT01001518	QT02270226
GPR26	QT02423778	QT00285215
GPR27	QT01001525	QT01062754
GPR31	QT00211442	NA
GPR32	QT01001546	NA
GPR34	QT01001553	QT00168938
GPR35	QT02403128	QT00495411
GPR37	QT00220759	QT01062747
GPR37L1	QT00023247	QT00157409
GPR39	QT00223216	QT00315028
GPR45	QT02423512	QT00258657
GPR50	QT00213346	QT00124117
GPR52	QT01153152	QT01071847
GPR55	QT00019124	QT01162931
GPR56	QT01341438	QT01060472
GPR61	QT02423785	QT00125755
GPR62	QT00211904	QT00302393
GPR63	QT00233597	QT00136920
GPR64	QT00026460	QT00142149
GPR65	QT01011136	QT00144774
GPR68	QT00246253	QT02392397
GPR75	QT00201089	QT02530157
GPR77	QT00243971	QT02532803
GPR78	QT00218540	NA
GPR82	QT00213304	QT00126784
GPR83	QT02423729	QT00104216
GPR84	QT00040124	QT00279888
GPR85	QT02423526	QT00281022
GPR87	QT02423792	QT01038856
GPR88	QT00031409	QT02263681
GPR97	QT00065653	QT01053227
GPR98	QT00025214	QT01061340
GPR101	QT01026928	QT00309512
GPR110	no primer*	QT00198170
GPR111	QT01193255	QT01162679
GPR112	QT01193094	QT02327584
GPR113	QT01678082	QT01168748

GPR114	QT00037884	QT01161699
GPR115	QT01665538	QT01753808
GPR116	QT01033046	QT01070272
GPR119	QT01030848	QT01758953
GPR123	QT01668310	QT01069404
GPR124	QT00005243	QT00129731
GPR125	QT01674134	QT01057294
GPR126	QT00048825	QT01065862
GPR128	QT00054824	QT02248449
GPR132	QT00243131	QT00249900
GPR133	QT00055937	QT01775907
GPR135	QT01192863	QT01040977
GPR137	QT00092848	QT00158263
GPR139	QT00229565	QT00306803
GPR141	QT01034943	QT01163561
GPR142	QT02423540	QT01040298
GPR143	QT00012635	QT00494613
GPR144	QT00073808	QT02303084
GPR146	QT00202041	QT00292726
GPR148	QT01034719	NA
GPR149	QT01034726	QT00123578
GPR150	QT01034068	QT00279293
GPR151	QT01030694	QT00281085
GPR152	QT00234486	QT00267477
GPR153	QT00096719	QT00117859
GPR156	QT00053431	QT00140784
GPR157	QT01151143	QT01061284
GPR158	QT00080738	QT01059142
GPR160	QT00047222	QT01075375
GPR161	QT01017345	QT01064399
GPR162	QT00065891	QT00098973
GPR171	QT00097958	QT01050700
GPR173	QT00047558	QT01164716
GPR174	QT01027691	QT01542695
GPR176	QT02423659	QT00126532
GPR179	QT00064323	QT02243598
GPR182	QT00228487	QT01163666
GPR183	QT00000441	QT00295785
GPRC5A	QT01153488	QT00104832
GPRC5B	QT00026628	QT00124348
GPRC5C	QT00057169	QT00148582
GPRC5D	QT02423568	QT00132363

GPRC6A	QT02423967	QT00147098
GRM1	QT02423463	QT00175042
GRM2	QT02423470	QT02327822
GRM3	QT02423484	QT00171542
GRM4	QT00060396	QT01072855
GRM5	QT00026502	QT01552117
GRM6	QT01001735	QT00133525
GRM7	QT00069727	QT01167509
GRM8	QT02423498	QT00169267
GRPR	QT00196875	QT00248885
HCAR1	no primer*	QT00258461
HCAR2	no primer*	QT00264509
HCAR3	no primer*	NA
HCRTR1	QT02423687	QT01755054
HCRTR2	QT02423981	QT00171430
HRH1	QT00199857	QT01161300
HRH2	QT02423694	QT01039157
HRH3	QT00210861	QT00158375
HRH4	QT02423869	QT00135884
HTR1A	QT02423848	QT00250516
HTR1B	QT01157653	QT01039913
HTR1D	QT00205618	QT01759156
HTR1E	QT01002666	NA
HTR1F	QT01002673	QT00102242
HTR2A	QT00054306	QT00282947
HTR2B	QT00060368	QT00144704
HTR2C	QT01002680	QT00144403
HTR4	QT00085624	QT00108143
HTR5A	QT00203000	QT00248290
HTR6	QT00201117	QT00261870
HTR7	QT02423722	QT00150346
KISS1R	QT00043134	QT00140427
LGR4	QT01529773	QT01065295
LGR5	QT00027720	QT00123193
LGR6	QT00085827	QT02525292
LHCGR	QT00081571	QT00101990
LPAR1	QT00021469	QT00107709
LPAR2	QT01851318	QT00106008
LPAR3	QT00092932	QT00264320
LPAR4	QT00235697	QT00125888
LPAR5	QT00209503	QT00312571
LPAR6	QT01842876	QT00325668

LPHN1	QT00015967	QT01048292
LPHN2	QT00006678	QT02284037
LPHN3	QT00007560	QT01066856
LTB4R	QT00097482	QT00197897
LTB4R2	QT02423736	QT01054935
MAS1	QT00204302	QT02257157
MAS1L	QT01029693	NA
MC1R	QT01004241	QT00305011
MC2R	QT01155007	QT01066338
MC3R	QT00209895	QT00264404
MC4R	QT00245595	QT00280861
MC5R	QT00211960	QT01166494
MCHR1	QT00213031	QT00312291
MCHR2	QT00098763	NA
MLNR	QT02423743	NA
MRGPRD	QT00237811	QT01078917
MRGPRE	QT00245357	QT00282205
MRGPRF	QT01032857	QT01078924
MRGPRG	QT01173235	QT00298480
MRGPRX1	QT00215649	QT02330237
MRGPRX2	QT00215656	QT02530808
MRGPRX3	QT00220094	NA
MRGPRX4	QT00221683	NA
MTNR1A	QT00034559	QT00253029
MTNR1B	QT01004647	QT01040648
NMBR	QT00099904	QT00312494
NMUR1	QT00080311	QT00174006
NMUR2	QT00032284	QT00137998
NPBWR1	QT00214480	QT00299040
NPBWR2	QT01001483	NA
NPFFR1	QT00041363	QT02528113
NPFFR2	QT00016877	QT00303975
NPSR1	no primer*	QT00152180
NPY1R	QT00081340	QT00249746
NPY2R	QT00008869	QT01744036
NPY5R	QT00231875	QT00109088
NTSR1	QT00018494	QT00163373
NTSR2	QT00022911	QT00165151
OPN3	QT00090657	QT00120218
OPN5	QT00029022	QT00140406
OPRD1	QT00000210	QT00103250
OPRK1	QT00015316	QT00101549

OPRL1	QT02423897	QT00300811
OPRM1	QT00001512	QT01770811
OXER1	QT00208845	NA
OXGR1	QT00051093	QT01160943
OXTR	QT00001715	QT01778427
P2RY1	QT00199983	QT00158340
P2RY2	QT01673147	QT00097202
P2RY4	QT00245623	QT02332764
P2RY6	QT01867670	QT00150010
P2RY8	QT00204876	NA
P2RY10	QT00038563	QT00494732
P2RY11	no primer*	NA
P2RY12	QT01155175	QT02527000
P2RY13	QT00246771	QT00124285
P2RY14	QT00199997	QT00173264
PPYR1	QT02423757	QT00128121
PRLHR	QT00211435	QT00326172
PROKR1	QT00030716	QT00314846
PROKR2	QT01030519	QT00157654
PTAFR	QT01673511	QT00262990
PTGDR	QT00036190	QT00114310
PTGDR2	QT00042448	QT00320537
PTGER1	QT00210070	QT00173936
PTGER2	QT00001330	QT00115276
PTGER3	QT02396170	QT00254303
PTGER4	QT02288314	QT02589440
PTGFR	QT00029540	QT00170562
PTGIR	QT00072807	QT00160062
PTH1R	QT00000672	QT01057784
PTH2R	QT00032543	QT00164647
QRFPR	QT00052388	QT02331175
RXFP1	QT00041720	QT00172382
RXFP2	QT00095725	QT00131124
RXFP3	QT00210133	QT00285222
RXFP4	QT01034453	QT01040375
S1PR1	QT00208733	QT00243628
S1PR2	QT00230846	QT00262773
S1PR3	QT00244251	QT00132160
S1PR4	QT01192744	QT00260141
S1PR5	QT00234178	QT00282744
SCTR	QT00009261	QT01066646
SMO	QT00050701	QT00494683

SSTR1	QT00029204	QT01761137
SSTR2	QT00081179	QT01539111
SSTR3	QT00215355	QT01162637
SSTR4	QT01681407	QT02264661
SSTR5	no primer*	QT01162630
SUCNR1	QT00048538	QT00127582
TAAR1	QT01030701	QT01041936
TAAR2	QT01012543	QT01066968
TAAR5	QT00214501	QT01064007
TAAR6	QT01034243	QT00360990
TAAR8	QT01674071	NA
TAAR9	QT00218323	QT01071490
TACR1	QT00028896	QT00103334
TACR2	QT00203616	QT00493199
TACR3	QT00000616	QT00134036
TAS1R1	QT00021014	QT00121870
TAS1R2	QT01026508	QT00142639
TAS1R3	QT00214270	QT00309890
TBXA2R	QT00066199	QT01160873
TRHR	QT00200109	QT00161931
TSHR	QT00025480	QT00136955
UTS2R	QT00213465	QT01040263
VIPR1	QT00044002	QT00167160
VIPR2	QT00073388	QT00106463
XCR1	QT01885611	QT00261310
ACTB	QT01680476	QT01136772
GAPDH	QT01192646	QT01658692
PPIA	QT01866137	QT00247709
TBP	QT00000721	QT00198443
TFRC	QT00094850	QT00122745
